# A Manual of Medicine

**Published:** 1904-06

**Authors:** 


**A Manual of Medicine.** Edited by W. H. ALLCHIN, M.D. Vol. V. Pp. xii., 687. London: Macmillan & Co. Ltd. 1903.

This volume of this useful manual deals with the diseases of the digestive system and of the liver, the diseases of the peritoneum and of the vessels of the abdomen, of the kidneys and those of the ductless glands. In discussing the diseases of these several organs, there are sections on the anatomy and physiology, on the pathology, general etiology, general and special symptomatology, and treatment of each. The book opens with a very useful section by the editor on the anatomy of the alimentary canal and on the physiology of digestion, and this is followed by a short but excellent practical chapter on food and diet by Dr. R. Hutchison. Dr. Lazarus Barlow contributes an interesting chapter on the bacteria of the alimentary canal. The diseases of the alimentary tract and their pathology then receive careful and adequate consideration from the editor, Dr. Bertram Abrahams, and Dr. Bertram Dawson. This contains a good section on the stomach, with directions for the examination of the gastric contents. The portion devoted to diseases of the pancreas is short, but probably contains as much as can be said with certainty at the present time on this difficult subject.

Dr. Hebb and the editor have written the section on diseases of the liver. In it will be found an excellent *resumé* of our present knowledge.

Dr. Hale White contributes a very able article on the diseases of the peritoneum, and this includes valuable sections on retro-peritoneal suppuration and tumours, and on subphrenic abscess. The chapters on diseases of the abdominal vessels, often so obscure and difficult of diagnosis, will be found very useful, and has been written by Dr. Bryant. Dr. Rose Bradford is responsible for the section on diseases of the kidneys. It is very well written, contains a large amount of information, and has the merit of clearness.

This volume well maintains the characters of the manual which we have pointed out in reviews of the preceding volumes. Anyone may read it with profit. It affords the practitioner the opportunity of possessing at a moderate cost an excellent modern work on medicine, which he may rely upon as a trustworthy guide, and it is not too long. Its moderate size precludes, of course, any lengthy consideration of a single subject and more than a brief notice of rare diseases, but the sections throughout are adequate, and present a faithful picture of modern medicine, and we can cordially recommend it as an excellent book for the student and a thoroughly good hand-book for reference in the everyday work of the practitioner. There are many good illustrations.

